# The role of the serotonergic system in locomotor recovery after spinal cord injury

**DOI:** 10.3389/fncir.2014.00151

**Published:** 2015-02-09

**Authors:** Mousumi Ghosh, Damien D. Pearse

**Affiliations:** ^1^The Miami Project to Cure Paralysis, University of Miami Miller School of MedicineMiami, FL, USA; ^2^Department of Neurological Surgery, University of Miami Miller School of MedicineMiami, FL, USA; ^3^The Neuroscience Program, University of Miami Miller School of MedicineMiami, FL, USA; ^4^The Interdisciplinary Stem Cell Institute, University of Miami Miller School of MedicineMiami, FL, USA

**Keywords:** serotonin receptor agonists, serotonin, spinal cord, locomotion control, central pattern generators (CPG)

## Abstract

Serotonin (5-HT), a monoamine neurotransmitter synthesized in various populations of brainstem neurons, plays an important role in modulating the activity of spinal networks involved in vertebrate locomotion. Following spinal cord injury (SCI) there is a disruption of descending serotonergic projections to spinal motor areas, which results in a subsequent depletion in 5-HT, the dysregulation of 5-HT transporters as well as the elevated expression, super-sensitivity and/or constitutive auto-activation of specific 5-HT receptors. These changes in the serotonergic system can produce varying degrees of locomotor dysfunction through to paralysis. To date, various approaches targeting the different components of the serotonergic system have been employed to restore limb coordination and improve locomotor function in experimental models of SCI. These strategies have included pharmacological modulation of serotonergic receptors, through the administration of specific 5-HT receptor agonists, or by elevating the 5-HT precursor 5-hydroxytryptophan, which produces a global activation of all classes of 5-HT receptors. Stimulation of these receptors leads to the activation of the locomotor central pattern generator (CPG) below the site of injury to facilitate or improve the quality and frequency of movements, particularly when used in concert with the activation of other monoaminergic systems or coupled with electrical stimulation. Another approach has been to employ cell therapeutics to replace the loss of descending serotonergic input to the CPG, either through transplanted fetal brainstem 5-HT neurons at the site of injury that can supply 5-HT to below the level of the lesion or by other cell types to provide a substrate at the injury site for encouraging serotonergic axon regrowth across the lesion to the caudal spinal cord for restoring locomotion.

## Introduction

Spinal cord injury (SCI) is a devastating condition affecting approximately 273,000 individuals in the US, with 12,000 new cases occurring annually (National Spinal Cord Injury Statistical Center, University of Alabama, https://www.nscisc.uab.edu). Damage to the spinal cord results in the impairment of specific functions controlled by the nerves located at, or below, the level of SCI. According to the presence or absence of motor function, human SCI can be classified as complete or incomplete. Following incomplete injury, a certain degree of movement and sensation below the level of injury may be retained depending upon the severity of the injury and the corresponding extent of axonal preservation. Complete SCI results in paralysis, due to the lack of sensory and motor function below the level of injury, though rarely is SCI anatomically complete in man. One of the major consequences resulting from trauma to the spinal cord is the disruption of the dynamic interactions among the spinal neuronal network, supraspinal pathways and peripheral sensory inputs, which results in an impairment of locomotor function (Kiehn, 2006; Rossignol et al., 2006). This dysfunction is permanent due to the subsequent loss of motor neuron excitability and the inability of the central nervous system (CNS) to mount a robust reparative response endogenously. Although the complete ablation of direct supraspinal projections to regions below the injury results in the cessation of the voluntary control of movements, some residual motor function may remain from the spontaneous reorganization and recovery of neuronal network excitability from plastic changes within any spared ascending and descending systems (Ghosh et al., 2009; Tansey, 2010; Martinez and Rossignol, 2011; Rossignol and Frigon, 2011; D’Amico et al., 2014; El Manira, 2014; Filli et al., 2014).

To repair the injured spinal cord and restore voluntary motor function various strategies have been employed. Among the different approaches used, those that have elicited the sprouting or re-growth of serotonergic fibers caudal to the site of SCI have often been associated with an ensuing improvement in locomotor function (Bregman et al., 2002; Engesser-Cesar et al., 2007). The reactivation of the central pattern generator (CPG), an important spinal cord center for locomotor output, and stimulation of the locomotor neural network through (1) the exogenous application of 5-HT (Cazalets et al., 1992; Madriaga et al., 2004; Thompson et al., 2011) or its precursor, L-5-hydroxytryptophan (5-HTP; Hayashi et al., 2010; Meehan et al., 2012); (2) through the administration of agonists for specific serotonergic receptor subsets, either alone (Antri et al., 2002; Landry and Guertin, 2004) or in combination with the stimulation of the dopaminergic (DA) and the noradrenergic (NA) systems (Brustein and Rossignol, 1999; Musienko et al., 2011) or; (3) the transplantation of serotonergic embryonic neurons that can innervate these regions (Privat et al., 1989; Rajaofetra et al., 1989a; Gimenez y Ribotta et al., 1998), have provided convincing evidence in support of an essential and indispensable role for the serotonergic system in promoting the restoration of locomotor function after SCI. Though it has been shown that concurrent stimulation of all the monoaminergic systems, NA, DA and serotonergic (Jordan et al., 2008), is important in the generation of locomotion, the present review will focus specifically on the function of the different components of the serotonergic pathway in regulating motor function output with attention to how these components are altered after SCI and lead to locomotor dysfunction. In addition, the therapeutic modalities that have been employed to modulate the serotonergic system in the CNS, in an attempt to restore locomotor function after experimental SCI, are discussed.

## 5-HT and anatomical localization of its neural pathways

5-HT is a monoamine neurotransmitter synthesized from tryptophan, an essential amino acid, by a subset of neurons referred to as serotonergic neurons that are present in the CNS as well as by enterochromaffin cells in the gastrointestinal tract (Li et al., 2014). The anatomical localization of 5-HT pathways in the CNS was initially delineated in the rat brain by Dahlstroem and Fuxe (1964), who demonstrated that serotonergic neurons were largely concentrated in the raphe nuclei of the brainstem. Almost all the 5-HT axons found within the mammalian spinal cord originate supraspinally from neurons located in the brainstem (Takeuchi et al., 1982), primarily from three main regions, the medullary raphe pallidus, raphe obscuris and the raphe magnus (Azmitia, 1999), as well as part of the reticular formation that encompasses the pyramidal tract at these levels. The 5-HT axon terminals originating from descending brainstem projections exist at all levels of the spinal cord (Rajaofetra et al., 1989b; Hornung, 2003) and are localized to the dorsal horn, ventral horn and intermediate area (Ballion et al., 2002). Serotonergic projections innervating the dorsal horn predominantly arise from the raphe magnus via the dorsolateral funiculus, which also has sparse projections in the ventral horn. The neurons in the raphe obscuris and pallidus project to the ventrolateral white matter as well as terminate onto motoneurons in the ventral horn and in the intermediate gray via the ventral and ventrolateral funiculi, respectively (Azmitia and Gannon, 1986). The axon collaterals from a single raphe neuron are able to innervate both the sensory and motor nuclei of the autonomic system at different spinal levels, including both cervical and lumbar regions of the spinal cord (Martin et al., 1981).

Previously, Rajaofetra et al. (1992a) demonstrated in adult baboons that serotonergic innervation of the Onuf’s nucleus, located in the ventrolateral part of the sacral spinal cord, was of both supraspinal and intraspinal origin. Supraspinal innervation was found to exist throughout the whole nucleus, with a predominance in the dorsal half, while the intraspinal innervation was primarily associated with the ventral half of the nucleus. Subsequently, Branchereau et al. (2002) reported in organotypic spinal cord cultures, which lacked descending serotonergic input, the expression of 5-HT in intraspinal neurons; these 5-HT intraspinal neurons were able to compensate for the lack of the descending supraspinal 5-HT fibers and contribute to the development of spontaneous locomotor activity. The 5-HT expressed by these intraspinal neurons was dependent upon the absence of 5-HT fibers as 5-HT from the descending input repressed expression of 5-HT from these intraspinal neurons. In the human spinal cord, Perrin et al. (2011) have mapped 5-HT profiles in the thoracic and lumbar segments, finding a similar neuroanatomical localization to that of rodents and non-human primates where serotonergic processes were identified primarily within the ventral horn surrounding motoneurons as well as also in the intermediolateral region and in the superficial part of the dorsal horn.

## Function of the serotonergic pathway in locomotion

Multiple descending tracts from the brainstem function in the initiation and regulation of locomotion, including the glutamatergic, NA, DA and 5-HT pathways. These functions are mediated through the action of various neurotransmitters such as glutamate, NA, 5-HT and DA, which induce spinal motor activity, stimulate rhythmic activity and control segmental reflexes (Humphreys and Whelan, 2012; Beliez et al., 2014; Sławińska et al., 2014a). The role of 5-HT in locomotion remains, however, only partially understood. Experimental work from a number of research laboratories have provided convincing evidence that 5-HT regulates the rhythm and coordination of movements through the CPG. The CPG constitutes a major anatomical component of locomotion comprised of neurons distributed within a neural network in the thoraco-lumbar spinal cord that drives motoneuron output to generate simple rhythmic patterns, such as locomotion (Grillner and Walleń, 1985; Kiehn and Kullander, 2004). 5-HT has been recognized as a potent neuromodulator of CPG activity (Feraboli-Lohnherr et al., 1997). The CPG in the lumbar spinal cord is regulated both by supraspinal descending inputs that originate in the raphe nucleus and terminate in the intermediate gray and the ventral horn (Carlsson et al., 1963; Ballion et al., 2002) as well as by sensory afferents (Rossignol et al., 1988).

The neuronal network of the CPG, in response to monoaminergic input from the brainstem, contributes to the regulation of postural muscle tone and locomotion by determining which components of the specific locomotor program are necessary at a required or specific moment with respect to velocity, magnitude and duration (Takakusaki et al., 2004). Under normal conditions, the different motor programs existing in the brainstem remain in a state of inhibition during rest (Grillner, 2006; Hikosaka, 2007). Fornal et al. initially suggested that under these conditions serotonergic neurons are controlled by tonic feedback inhibition (Fornal et al., 1994). When locomotion is initiated, signals activating the locomotor network in the mesencephalic locomotor region (MLR) and the diencephalic locomotor region (DLR) of the midbrain converge on reticulospinal neurons in the brainstem, which determine the extent and duration of locomotor activity that is necessary to be generated by the CPG. Therefore, the MLR, when subjected to electrical or chemical stimulation, triggers bouts of locomotion and elicits movement by activating the reticulospinal pathways (Shik et al., 1966; Garcia-Rill et al., 1985; Sholomenko and Steeves, 1987; Steeves et al., 1987). The initial synaptic targets of the MLR are neurons in the medial pontomedullary reticular formation (MdRF), after which their axons descend as the reticulospinal tract within the ventrolateral funiculus of the spinal cord to synapse on CPG neurons within the cervical or lumbar segments (Steeves and Jordan, 1984; Garcia-Rill et al., 1985; Noga et al., 1991).

As first discussed by Jacobs and Fornal (1993), the primary function of 5-HT neurons in the brainstem is to facilitate motor output during periods of tonic motor activity, such as postural shifts, or to control repetitive motor behaviors that are mediated by the spinal cord CPGs, such as locomotor speed. However, when the spinal cord is injured, there can be significant disruption or complete severing of the serotonergic projections, as well as other descending systems, to the CPG and loss of locomotor output. When exogenous 5-HT or selective 5-HT receptor agonists are supplied systemically or intraspinally after SCI, in combination with sufficient excitation by epidural electrical stimulation (EES) or glutamate, these locomotor behaviors can be re-elicited (Schmidt and Jordan, 2000; Antri et al., 2002; Landry et al., 2006; Courtine et al., 2009; Fouad et al., 2010).

5-HT participates directly in modulating motor function output through its binding to specific 5-HT receptors present upon the membrane of motoneurons. Depending on the specific receptor subtypes that are activated, either depolarization or hyperpolarization of the motoneurons occurs—thus 5-HT acts a control point in the regulation of spinal motoneuron excitability, leading to an amplification of synaptic excitation or inhibition (Perrier et al., 2013). Early studies employing selective 5-HT receptor agonists and antagonists provided evidence that the activation of 5-HT_1A_ receptors could mediate inhibitory responses, whereas excitation was produced by the activation of 5-HT_2A_ receptors (Bayliss et al., 1995). Excitatory neurotransmission by 5-HT occurs through the modulation of various ion channels which leads to a sustained depolarization due to the presence of persistent inward currents that are mediated by voltage sensitive Ca^2+^ and Na^+^ conductance to cause an amplification of synaptic input. Following SCI, there is a reduced input of brain stem-derived 5-HT which results in an altered membrane potential of the motoneurons causing an acute suppression of motoneuron excitability. This is attributed to various factors such as motoneuron hyperpolarization coupled to an acute disappearance of voltage-activated sodium and calcium persistent inward currents which prevents activation of action potentials. This inhibits motoneuron firing and increases pre-synaptic inhibition thereby rendering the motoneuron and spinal neuronal circuitry unexcitable acutely after the injury. 5-HT can also modulate motoneuron excitability indirectly through its effects on spinal interneurons where 5-HT can alter the action potential properties of these interneurons, especially following SCI. It has been observed in mouse lumbar V2a spinal interneurons (Husch et al., 2012) that an enhanced 5-HT super sensitivity occurs after SCI due to the elevated density of 5-HT_2C_ receptors on the cell membrane without promoting any significant changes in their level of excitability. 5-HT thus participates in regulating the firing frequency and excitability of spinal motor neurons, which corresponds to its ability to control the speed and amplitude of locomotion as well as alter the membrane properties of spinal interneurons (Harris-Warrick and Cohen, 1985; Zhang and Grillner, 2000; D’Amico et al., 2014; Wienecke et al., 2014) to promote increased motoneuron excitability. SCI-induced losses of 5-HT, however, can lead not only to an absence of motoneuron excitability and locomotor output but residual 5-HT or activity of its receptors may also produce aberrant motoneuron excitability that is involved in triggering spasticity and/or impaired motor output that can hinder normal locomotion (Perrier et al., 2013). This is an outcome observed following chronic SCI, where persistent inward currents are enhanced either due to compensatory over-expression of spontaneously active 5-HT_2_ receptors (Murray et al., 2010) or as a result from a depolarized chloride reversal potential (Boulenguez et al., 2010).

## 5-HT receptor activation and locomotion

The different members of the 5-HT family of receptors are located within distinct areas of the central and the peripheral nervous systems, as well as in non-neuronal tissues, and are involved in a diversity of functions. The 5-HT receptor family represents one of the most complex families of neurotransmitter receptors that have been characterized to date. Studies conducted by a number of research groups have provided convincing experimental evidence that the descending serotonergic system modulates spinal reflexes and motor function/hind limb coordination through the activation of specific 5-HT receptors, which in turn cause an increase in motoneuron and interneuron excitability and the generation of CPG-mediated locomotor output (Schmidt and Jordan, 2000; Hochman et al., 2001; Pflieger et al., 2002).

A number of 5-HT receptor subtypes are expressed in high density on the membranes of motoneurons, including 5-HT_1A_, 5-HT_1B_, 5-HT_1D_, 5-HT_2A_, 5-HT_2B_, 5-HT_2C_ and 5-HT_5A_ (Perrier et al., 2013) as well as some spliced variants, 5-HT_3_, 5-HT_4_, 5-HT_6_, 5-HT_7_ and RNA edited isoforms, such as 5-HT_2C_ (Werry et al., 2008). These receptors mostly exist as homodimers, although they may also undergo heterodimerization (Renner et al., 2012). Dimerization occurs during their biosynthesis within the endoplasmic reticulum, which is important for their subsequent transport and expression in the plasma membrane as well as maximizes downstream coupling with the G-protein subunits (Herrick-Davis, 2013) that leads to motoneuron excitability. Delineating the signaling mechanisms regulated by 5-HT has been difficult due to both the existence of multiple 5-HT receptor isoforms that are activated in the presence of 5-HT as well as the unavailability of highly selective pharmacological modulators that can act on single receptor subtypes. In the human brain, 13 5-HT GPCRs and one serotonin-gated ion channel receptor, 5-HT_3_, have been identified (Millan et al., 2008; Lambe et al., 2011). Though 5-HT receptors have been classified under seven known subfamilies, 5-HT_1_ through 5-HT_7_, post-genomic modifications, such as alternative mRNA splicing or mRNA editing, have resulted in the identification of at least 30 distinct 5-HT receptor subtypes (Raymond et al., 2001). It has been demonstrated that the combined activation of more than one 5-HT receptor subtype is required for the generation of locomotor output, with signaling originating primarily from 5-HT_1A_, 5-HT_2A/C_ and 5-HT_7_ receptor subtypes (Jordan et al., 2008). Work to date in characterizing 5-HT receptor activation involvement in real or fictive locomotor output have focused largely on motoneurons, as they are the direct effecters of locomotor activity, in addition to the spinal interneurons that comprise the CPG.

With the availability of agonists and antagonists that exhibit greater selectivity for specific 5-HT receptor subtypes, studies have begun to identify which 5-HT receptor subtypes mediate specific locomotor behaviors. Employing receptor-specific agonists and antagonists, which have varying binding affinities towards each of the 5-HT receptor subsets, researchers have identified 5-HT_1A_, 5-HT_2A/2C_ (Courtine et al., 2009), 5-HT_3_ (Guertin and Steuer, 2005), and 5-HT_7_ receptors (Liu et al., 2009) as important players in the regulation of the spinal locomotor network and which are able to generate locomotion in experimental models of SCI when employed in conjunction with the activation of other monoaminergic systems (Kim et al., 2001a; Antri et al., 2003; Fuller et al., 2005; Madriaga et al., 2004; Guertin and Steuer, 2005; Ung et al., 2008; Courtine et al., 2009; Liu et al., 2009; Musienko et al., 2011). Studies employing 5-HT receptor selective agonists and antagonists in rats following spinal transection have identified that 5-HT_2_ receptors are predominantly responsible for mediating the depolarizing effects resulting from decreased potassium conductance in response to a 5-HT stimulus (Jacobs and Fornal, 1993; Harvey et al., 2006; Li et al., 2007; Perrier et al., 2013; Gackière and Vinay, 2014). In experiments carried out by Ung et al. (2008), in which behavioral and kinematic analyses were performed at 1 week after complete spinal cord transection in mice, they identified the involvement of specific 5-HT_2_ receptor subtypes in locomotor-like movements through the use of selective antagonists. The non-selective 5-HT_2_ receptor agonist quipazine was able to induce locomotor-like movements in the presence of the selective 5-HT_2_ antagonists SB204741 and SB242084, which are inhibitory towards 5-HT_2B_ or 5-HT_2C_, respectively. In contrast, quipazine failed to induce locomotor-like movements in animals that had been pretreated with MDL-100,907, a selective 5-HT_2A_ antagonist. This work provided evidence that 5-HT_2A_ receptors were involved in spinal locomotor network activation and the generation of locomotor-like movements induced by quipazine in transected animals. Antri et al. (2003) showed that the combined, daily stimulation of 5-HT_2_ and 5-HT_1A_ receptors, using quipazine and 8-OHDPAT, respectively, after thoracic spinal cord transection in rats was more potent in restoring locomotion then when the 5-HT_2_ agonist was employed alone, suggesting a cumulative effect of activating the two receptor subtypes in facilitating locomotion. 5-HT_1A_ receptors are localized throughout the CNS with high expression in limbic regions and in the dorsal raphe nucleus, while in the spinal cord, 5-HT_1A_ receptors are identified primarily within the ventral horn surrounding motoneurons, although 5-HT_1A_ receptors are more densely expressed at the lumbar level of the spinal cord. 5HT_1A_ receptors have been shown to produce hyperpolarization preferentially in a small subset of neurons, such as CA1 hippocampal neurons (Bobker and Williams, 1990; Levkovitz and Segal, 1997) and interneurons (Segal, 1990a,b). Therefore depending upon the specific receptor that 5-HT binds to, it can elicit either depolarizing or hyperpolarizing effects.

## Caudal serotonergic axon innervation and 5-HT levels after SCI and their effects on recovery

With 5-HT release within the spinal cord ventral horn playing a major role in mediating locomotor function, it is not surprising that following SCI, when there is severing of supraspinal serotonergic projections and depletion of 5-HT (Carlsson et al., 1963), that loss of 5-HT is one of the major limiting factors that prevents the recovery of motor function (Hashimoto and Fukuda, 1991). 5-HT loss after SCI is characterized by significant reductions or an absence of important enzymes such as tryptophan hydroxylase (TPH), which catalyzes the conversion of tryptophan to 5-hydroxytryptophan (5-HTP), that are necessary to generate 5-HT (Clineschmidt et al., 1971). Loss of 5-HT prevents the activation of the spinal locomotor CPG, interfering with the ability to evoke normal locomotion. When 5-HT levels are restored, locomotor function after SCI has been shown to be improved (Pearlstein et al., 2005).

Previous work by Hentall et al. (2006) characterized the spatial and temporal patterns of 5-HT release in the rodent lumbar spinal cord following electrical stimulation of the raphe magnus in SCI rats subjected to acute spinal cord transection at T6–T7. Their findings indicated that the action of monoamines in the spinal cord involves a combination of both synaptic neurotransmission as well as non-synaptic diffusion, similar to that previously observed (Bach-y-Rita, 1999). Noga et al. (2004) employed fast cyclic voltammetry, capable of measuring monoamines such as 5-HT at high spatial resolution in comparison to microdialysis-HPLC, to map the basal and steady-state extracellular distribution of monoamines released from tonically active, descending nerve terminals in the lumbar spinal cord of rats when at rest and after spinal cord transection. Their results demonstrated that at rest there was a greater concentration of 5-HT localized in specific regions of the dorsal and ventral horn of the lumbar cord as well as in the lateral region of the intermediate zone which varied within the different segments of the lumbar cord. Following thoracic spinal cord transection, a transient injury evoked increase in monoamine levels was noted briefly acutely within the distal stump of the transected cord. Maximal levels were measured in the superficial dorsal horn of the lumbar cord during this transient period after which there was a steady longer lasting decrease in monoamine levels (Noga et al., 2004). Recently, Gerin et al. (2010) examined temporal alterations in the level of endogenous 5-HT release in the ventral horn of rats that were subjected to a sub-hemi-section of the spinal cord at the T9 vertebral level. Release of 5-HT was measured using a microdialysis probe and HPLC analysis, with results showing variations in the levels after SCI over a period of a month during the post-injury recovery period as well when the injured animals were subjected to treadmill exercise. It was found that a significant decrease in 5-HT levels occurred after SCI, which was <50% of that observed in non-lesioned animals, indicative of serotonergic denervation. With time post-SCI, 5-HT levels were found to rise, increasing by 202% at 18 days post injury compared to the levels observed at 8 days post-lesion, which correlated with the timing of motor function recovery. When treadmill exercise was performed, a ~10% decrease in 5-HT levels was observed compared to rest at 18 and 34 days post-SCI.

## Alterations in 5-HT receptor activity after SCI

Previous studies using a complete spinal transection in the cat have shown that SCI leads to an immediate and dramatic reduction in motoneuron excitability (Hounsgaard et al., 1988; Miller et al., 1996). Spinal cord contusion in rodents, an incomplete SCI paradigm that is more clinically-relevant, leads to the preservation of a small number of serotonergic fibers that can undergo sprouting with time following an injury. The degree of locomotor function restoration observed after SCI has been reported to correlate to the density of sprouting of residual, uninjured serotonergic axons (Holmes et al., 2005; Nardone et al., 2014). The use of 5-HT receptor agonists in animals after an acute spinal transection has been shown to produce restoration of motoneuron excitability (Harvey et al., 2006; Li et al., 2007; Musienko et al., 2011; Sławińska et al., 2014a). Work by various researchers have demonstrated that locomotor-activated spinal neurons, which constitute the CPG network, are innervated by serotonergic fibers (Johnson et al., 2002; Perrin et al., 2011; Sławińska et al., 2014b) and that 5-HT is released within these sites during locomotor activity (Gerin et al., 2008). Together, these results have shown that serotonergic innervation and the availability of 5-HT in the lumbar cord (Antri et al., 2002) are critical for the restoration of hind limb motor function following SCI.

The loss of 5-HT can be overcome through the direct activation of 5-HT receptors, an approach which has been convincingly associated with the induction of locomotor output following SCI (Brustein and Rossignol, 1999; Feraboli-Lohnherr et al., 1999; Kim et al., 2001b). With the reduction or loss of 5-HT input caudal to the injury in rodent models of SCI, it has been demonstrated that there is a compensatory over-expression of 5-HT receptors (Fuller et al., 2005; Navarrett et al., 2012; Ren et al., 2013). In the lamprey, a vertebrate that exhibits locomotor recovery after complete SCI, a transient over-expression of the 5-HT_1A_ receptor transcript is observed for several weeks after SCI (Cornide-Petronio et al., 2014). In rats, Kong et al. (2010) reported a dramatic increase in 5-HT_2A_ receptor immunoreactivity in motoneurons caudal to the site of spinal cord transection, beginning 1 day after injury and persisting to maximal expression by 28 days which paralleled a concurrent reduction in the levels of 5-HT and the 5-HT transporter (Kong et al., 2011; Husch et al., 2012). Work by Otoshi et al. (2009) examined the influence of descending serotonergic input and sensory feedback on spinal 5-HT receptor expression using two paradigms. The first was a complete thoracic (T7–T8) spinal cord transection in adult rats, to eliminate supraspinal 5-HT input, while the second employed animals subjected to spinal cord isolation, so as to completely ablate both descending supraspinal as well as peripheral sensory input, completely eliminating any neuromuscular activity. They found that there was an enhanced expression of the 5-HT_1A_ receptor after transection that was dependent upon the presence of sensory input.

Murray et al. (2010) have provided findings that may explain how motoneurons are able to mediate some residual hind limb function in rodent models of complete spinal cord transection (S2, sacral spinal level) or with staggered hemisection, both of which remove the descending supraspinal projections caudal to the level of SCI. It appears that this residual function can arise as a result of alterations in 5-HT receptor expression and their constitutive activation as a compensatory mechanism. A SCI-induced alteration in the levels of the 5-HT_2C_ receptor has been correlated to a change the post-transcriptional editing of its mRNA that results in both elevated production and constitutive activation. SCI-mediated constitutive activation of 5-HT_2C_ receptors is able to restore large persistent calcium currents in motoneurons, which in turn is able to induce sustained muscle contractions and the restoration of motor function. Therefore, despite the non-availability of a serotonergic stimulus following SCI, which results in loss of motoneuron excitability acutely, a compensatory upregulation of spontaneously active 5-HT_2_ receptors induced at chronic stages after SCI appears to be an adaptive mechanism which could be responsible for residual locomotor function.

The use of EES, applied onto the dorsal surface of the lumbar spinal cord or given intraspinally, has been used in decerebrated and acute spinal cord transected cats (Iwahara et al., 1992; Gerasimenko et al., 2007) as well as in rats (Ichiyama et al., 2005) to activate the spinal locomotor circuits so as to induce hind limb movements and locomotion (Gerasimenko et al., 2007; Courtine et al., 2009). The use of EES in SCI patients, to promote locomotor function, was first employed by Dimitrijevic et al. (1998), later optimized in paralyzed individuals in work by Shapkova et al. (Shapkova and Schomburg, 2001; Shapkova and Mushkin, 2002; Shapkova, 2004) and is currently being evaluated in clinics in the US (Harkema et al., 2011). Recently, Angeli et al. (2014) successfully used epidural stimulation to activate spinal circuitry in completely paralyzed American Spinal Injury Association Impairment Scale (AIS) A patients, opening a new avenue for therapeutic interventions to promote functional recovery after SCI. Through the use of immunohistochemistry, Noga et al. (2009) showed the co-localization of serotonergic axons with specific 5-HT receptor subtypes 5-HT_1A_, 5-HT_2A_ and 5-HT_7_, on c-Fos immunoreactive neurons within laminae VII and VIII of the thoraco-lumbar spinal cord segments following EES in pancuronium-paralyzed, decerebrate cats. The presence of these 5-HT receptor subtypes on these spinal neurons in T13–L7 spinal cord indicates that they likely play a role in 5-HT’s modulation of locomotion.

To assess the roles of the specific 5-HT receptor subtypes after SCI that have been identified to be involved in locomotor output function; 5-HT_1A_ (Courtine et al., 2009), 5-HT_2A/2C_ (Courtine et al., 2009), 5-HT_3_ (Guertin and Steuer, 2005) and 5-HT_7_ (Liu et al., 2009), Musienko et al. (2011) conducted experiments using pharmacological manipulations with combinations of receptor specific agonists and/or antagonists. These studies were performed in adult rats subjected to a complete spinal cord transection at the mid-thoracic vertebral level (T7) and the effects of receptor manipulations on observed functional recordings were analyzed during locomotion enabled by EES. From these studies, specific functional roles for each of the 5-HT receptors in modulating motor function output were identified. It was shown that when transected animals were pre-treated with the 5-HT_7_ specific antagonist SB269970, prior to the administration of 8-OHDPAT, a dual agonist for 5-HT_1A/7_, and used in conjunction with EES, a marked improvement in the animal’s stepping patterns and inter-limb coordination was observed along with a reduction in kinematic variability and an increase in weight-bearing capacity. These improvements were not seen when EES was used alone, demonstrating an essential role of 5-HT_1A_ receptors in facilitating locomotion, as also reported by other groups (Antri et al., 2003; Courtine et al., 2009). Similarly, they observed that activation of 5-HT_2A/C_ receptors with the general 5-HT_2_ receptor agonist quipazine, in the presence of EES, produced facilitation of extension of the distal joints along with an improvement in weight-bearing capacity that was not observed with EES alone. When the 5-HT_3_ receptor agonist SR57227A was used with EES, the animals exhibited both an enhancement in weight-bearing as well as a reduction in gait timing variability and paw dragging. The combined activation of 5-HT_7_ and 5-HT_1A_ receptors with EES also elicited an improvement in stepping pattern and produced a potent facilitation in joint flexion, while combined activation of 5-HT_7_ and 5-HT_2A/C_ receptors improved inter-limb coordination, reduced the duration of dragging and substantially increased weight bearing capacity. Finally, the simultaneous activation of all four receptor subtypes, 5-HT_1A_, 5-HT_2A/C_ and 5-HT_7_, with EES led to a significant increase in the EMG activity of the proximal extensor and flexor muscles compared with the stepping pattern elicited by EES alone. These results suggest that it may be necessary to target multiple 5-HT receptors in combination to obtain maximal efficacy in overall locomotor output in the presence of EES (Musienko et al., 2011), although it remains to be tested whether altering the sequential stimulation of different receptor subtypes will be needed to obtain optimal locomotor output.

In addition to pharmacological modification of specific 5-HT receptor subtypes, more recent work has focused on the use of molecular tools to examine receptor function using knockout approaches in transgenic animals. In mice lacking the 5-HT_1A_ receptor, work by Scullion et al. (2013) confirmed the important role of 5-HT_1A_ receptors in the generation of locomotion. 5-HT_1A_ knockout mice showed higher movement thresholds and smaller motor maps compared with wild-type animals. Increased use of transgenic animals, particularly conditional knockouts, to study the role of specific receptor subtypes in the spinal cord should be undertaken to provide a more specific approach than pharmacological agents for dissecting the function different 5-HT receptors play in the generation of motor output, both under normal locomotion and in the context of locomotor recovery following SCI.

## Differential activity of 5-HT receptor subtypes in inducing locomotion after SCI

Although 5-HT acts on its multiple receptor subtypes, it can trigger different signaling pathways that may have excitatory (Cazalets et al., 1992; MacLean et al., 1998) or inhibitory (Schmidt and Jordan, 2000) effects on motor output. Depending on which 5-HT receptors are expressed and activated as well as their cellular and/or specific regional localization within the injured spinal cord, the serotonergic system can produce differential effects on locomotion (Landry et al., 2006; Courtine et al., 2009). Such a variation in response has been noted in phrenic motoneurons where 5-HT displays a differential effect elicited by the 5-HT_1B_ and 5-HT_2A_ receptor subtypes, where by one acts in a stimulatory capacity and the other is inhibitory (Holtmanj et al., 1986; Lalley, 1986). Work by Di Pasquale et al. (1997) showed that while 5-HT_2A_ activation mediated neuronal depolarization of phrenic motoneurons, pharmacological activation of the 5-HT_1B_ receptor resulted in the opposite effect, with inhibition of phrenic activity. This inhibition could be prevented by pre-treatment with 5-HT_1B_ receptor specific antagonists, indicating that opposite effects could be evoked in response to the activation of these two receptor subtypes. Thus 5-HT is able to regulate phrenic motoneuron firing via activation of spinal postsynaptic 5-HT_2A_ and presynaptic 5-HT_1B_ receptors, which in turn function to protect the diaphragm from overstimulation during a non-respiratory action such as vomiting or defecation. Similar differential effects of 5-HT elicited by different 5-HT receptor subtypes have also been demonstrated in spinalized rats, where monosynaptic transmission is inhibited or stimulated with the activation of 5-HT_1A_ and 5-HT_2A/2C_ receptors, respectively (Hasegawa and Ono, 1996). Using specific agonists for 5-HT_1_ and 5-HT_2_ receptor subtypes, differential effects on hind limb locomotor activity in adult mice after complete thoracic spinal cord transection were reported (Landry and Guertin, 2004). While 5-HT_2A/2C_ receptor agonists facilitated locomotion, stimulating 5-HT_2B/2C_ or 5-HT_1B_ receptors not only failed to induce locomotor-like movements, but also abated the hind limb movements induced by the non-specific 5-HT receptor agonist quipazine, when combined with L-DOPA. The involvement of 5-HT_2_ receptors in mediating excitation has also been shown in the completely transected cat spinal cord where restoration of extensor reflex excitability could be achieved only with 5-HT_2A/2C_ specific agonists in contrast to 5-HT_1A_ and 5-HT_1B/D_ receptors, which were determined to be ineffective (Miller et al., 1996).

## SERTs and locomotion after SCI

Neurotransmission between two or more cell types occurs primarily through volume or synaptic transmission where, with the former, the point of release of the neurotransmitter might be at a distant location from that of the target cells. Volume transmission is associated with nonsynaptic interneuronal communication involving a widespread activation of extrasynaptic as well as intrasynaptic receptors (Agnati et al., 1986). This produces a longer duration in the transmission of the signal and requires the diffusion of the neurotransmitter. In comparison, synaptic transmission occurs within a relatively constrained region of the synaptic cleft, between the pre- and the post-synaptic neurons. The duration of 5-HT signaling is regulated by the function of two types of transporters. The vesicular monoamine transporter (VMAT) which pumps 5-HT from the cytoplasm into synaptic vesicles and thereby controls the release of 5-HT from the presynaptic neurons. The other is the serotonin re-uptake transporter (SERT) which exists in the presynaptic plasma membrane of the serotonergic neurons and re-absorbs extracellular 5-HT back into the cytoplasm (Iversen, 1971; Blakely et al., 1991; Rudnick and Clark, 1993; Torres et al., 2003; Murphy et al., 2004) resulting in termination of 5-HT neurotransmission and preventing extrasynaptic transmission (Blakely et al., 1991; Schloss et al., 1992; Lesch et al., 1993). The regulation of serotonergic signaling by SERTs plays a key role in the regulation and the functioning of the serotonergic system in locomotion (Hayashi et al., 2010; Husch et al., 2012).

Various allosteric modulators of SERTs are currently gaining significant clinical importance as a way to enhance the functional capacity of these transport proteins when concentrations of 5-HT are rate limiting, such as observed following SCI. Work conducted by Hayashi et al. (2010) has suggested that even if 5-HT receptors are up-regulated following spinal cord transection and are thus available for the generation of locomotion when activated by specific agonists, the same approach may not be as effective in improving locomotor function in incomplete SCI models, such as a spinal cord contusion. Though spared supraspinal serotonergic projections are retained after incomplete SCI, there appears to be an absence of 5-HT receptor activation, despite an enhanced upregulation of 5-HT_2C_ receptors caudal to the injury. It was shown in this study that the administration of selective 5-HT_2C_ or 5-HT_1A_ receptor agonists, either alone or in combination after contusive SCI, was unable to produce receptor activation as evidenced from a lack of improvement in hind limb motor function. An improvement in motor function was only observed when there was broad receptor activation produced by the administration of the 5-HT precursor, 5-hydroxytryptophan (L-5-HTP), suggesting that an elevation in the levels of serotonergic receptors alone may not be sufficient to promote an improvement in motor function in models of incomplete injury. Even though there are spared serotonergic axons that are retained after contusive SCI, it has been suggested that they may have impaired function due to a dramatic reduction in the levels of SERTs, resulting in a dysregulation of 5-HT reuptake (Husch et al., 2012). Therefore prominent loss of SERT may be the causative reason for a reduced ability of the 5-HT agonists to improve locomotor outcome after an incomplete injury, indicating SERT as a critical limiting factor that is necessary to overcome locomotor impairment following SCI. Work to transgenetically overexpress SERTs in animals receiving SCI and the administration of selective 5-HT_1_ and 5-HT_2_ agonists would provide strong evidence for a role of SERTs in both SCI-mediated locomotor dysfunction and as a therapeutic modality for facilitating locomotor output.

## Transplantation paradigms involving the delivery of 5-HT expressing cells to improve locomotor outcome after SCI

Although a number of pharmacological approaches have been employed to activate the CPG to induce locomotion in models of SCI with varying degrees of success as described above, an alternative has been to introduce cells into the injured spinal cord that are capable of producing 5-HT. These exogenous 5-HT expressing cells can be used to replace the loss of monoaminergic supraspinal afferents and serve as a relay to restore serotonergic projections caudal to the level of the injury (Bregman, 1994). One such cell type that has been employed for this purpose is embryonic brainstem neurons or raphe neurons, which are able to extend serotonergic axons into adjacent host spinal tissue to deliver 5-HT following transplantation at, or below, the level of the injury (Privat et al., 1989; Rajaofetra et al., 1989a, 1992b; Gimenez y Ribotta et al., 1998; Ribotta et al., 2000). The intraspinal transplantation of these cells has been shown to reverse SCI-induced changes in 5-HT receptor densities and to restore hind limb weight support and locomotor activity on a treadmill, without the need for additional pharmacological interventions (Gimenez y Ribotta et al., 1996). Studies carried out by Feraboli-Lohnherr et al. (1997), employing the transplantation of embryonic brainstem neurons within the transected thoracic cord of chronically injured rats, showed that these neurons were able to provide serotonergic innervation to the lumbar cord. The projections from the transplanted brainstem neurons successfully activated the CPG to facilitate locomotor activity and this effect was further improved when a 5-HT re-uptake inhibitor was introduced. Majczyński et al. (2005) performed grafting of embryonic raphe nuclei cells into the spinal cord at T12, below the level of a complete spinal cord transection at T9 in adult rats at 1 month post injury and showed that hind limb locomotor function could be restored. The transected animals receiving the cell grafts, when placed on a treadmill, were able to walk with regular, alternating hind limb movements and exhibited plantar contact with the ground during the stance phase as well as ankle dorsiflexion during the swing phase of each step cycle. These behaviors were not observed in the injured control animals that did not receive the grafts and reflected the establishment of new serotonergic innervation to the caudal cord by the grafted neurons. The observed improvement in locomotor activity with embryonic raphe nuclei cell grafts could be reduced in the presence of the 5-HT_2_ antagonist cyproheptadine, and the loss of function regained with the 5-HT_2_ agonist quipazine, suggesting a 5-HT_2_ receptor mediated effect.

A similar improvement in locomotor performance was observed when embryonic raphe cells were transplanted into the spinal cord of chronically injured rats that had received a transection SCI at either T9 or T11 (Gimenez y Ribotta et al., 1996). The degree of locomotor recovery correlated to the level at which the SCI or the transplant were made, with a better outcome observed when these were performed at the T11 level, rather than T9, due to the closer proximity to the lumbar CPG and a greater degree of serotonergic axon reinnervation of the lumbar cord. Later work showed that with this approach, greater efficacy for restoring locomotor activity could be obtained when embryonic serotonergic neuron transplantation after SCI was performed even closer to the CPG, at the level of lumbo-sacral spinal cord (Orsal et al., 2002). In addition to using brainstem neurons, Eaton et al. (2008) used the transplantation of hNT2.19 cells, a human neuronal cell line differentiated to serotonergic neurons capable of secreting 5-HT, into the severely contused spinal cord of rats 1 week after SCI to restore serotonergic neurotransmission. When employed with the combination of environmental enrichment, a significant improvement in locomotor function was observed, including reductions in foot exo-rotation and footfall errors on a gridwalk test. Recently, Sławińska et al. (2013) intraspinally grafted E14 fetal neurons obtained from the medulla, containing the B1, B2 and B3 serotonergic regions, which constitutes all the likely sources of descending 5-HT tracts from the caudal brainstem, after SCI. These fetal neurons were grafted at the T10/11 vertebra level of adult rats with a complete spinal cord transection at the T9/10 level and were shown to successfully restore coordinated plantar stepping and improve intra- and interlimb coordination. Using pharmacological antagonists, it was further demonstrated that the transplants mediated their effects through the activation of 5-HT_7_ and 5-HT_2_ receptors localized to different populations of spinal neurons. Furthermore, even when these neurons were transplanted at 1 month post-SCI there was a restoration of coordinated hind limb function evoked in response to tail stimulation.

Studies employing cellular therapies to replace lost 5-HT projections to regions below the level of the lesion, particularly to the CPG, provide a promising means for restoring locomotion after SCI. At the same time, this approach may be able to overcome some of the limitations that might be anticipated with a pharmacological based strategy, such as a lack of persistent activation of 5-HT receptors as a result of receptor desensitization to prolonged agonist exposure (Kelly et al., 2008) or side effects associated with the systemic administration of these drugs (Sławińska et al., 2013). Studies have begun to examine feasible sources of serotonergic neurons that could be used for transplantation paradigms, particularly the use of induced pluripotent stem cell (IPSC) and transcription factor cocktails for the generation of these neurons from either fibroblasts or embryonic stem cells (Shimada et al., 2012). It remains to be determined whether these cells can be used to promote improved locomotor output after SCI when employed in the same experimental paradigms where embryonic brainstem neurons have demonstrated benefit.

## Cell therapeutic strategies to promote regeneration of serotonergic axons and functional recovery after SCI

Though there are adaptive mechanisms of endogenous plasticity within the injured spinal cord and reorganization at the level of the brain that compensates for the loss of transmission from multiple descending pathways (Hawthorne et al., 2011; Zörner et al., 2014), with severe injuries such mechanisms are not able to reverse paralysis and restore locomotor function without the use of additional therapeutic interventions. In experimental SCI paradigms a range of therapeutic approaches have been shown to limit tissue damage, promote axon growth and plasticity and improve the degree of functional recovery (Pearse et al., 2004; Boulenguez and Vinay, 2009). Of the most effective approaches to improve locomotor outcome after SCI, many have demonstrated a significant enhancement of growth and regeneration of descending serotonergic axons caudal to the lesion site (Menei et al., 1998; Tobias et al., 2003; Pearse et al., 2004; Ramer et al., 2004; Oatway et al., 2005; Boido et al., 2009; Ghosh et al., 2012; Hodgetts et al., 2013; Hou et al., 2013; Kanno et al., 2014). It has been shown in different SCI models, in mice and rats, that there is some degree of endogenous axon growth/sprouting of serotonergic axons that occurs (Bregman et al., 1997; Ramón-Cueto et al., 1998), though the extent of such growth is only short distance (Bregman et al., 1997) unless an exogenous strategy is employed. The use of cell therapeutic approaches (Tetzlaff et al., 2011), such as the implantation of olfactory ensheathing cells (OECs) in rats with a complete transection SCI and Schwann cell (SC) bridging, has shown that significant long distance regeneration of serotonergic axons across a bridged SCI gap and into the contiguous caudal cord can be obtained (Ramón-Cueto et al., 2000); similar findings with OECs have been reported in other SCI paradigms (Lu et al., 2002; Plant et al., 2003). Though rats receiving OECs did exhibit modest improvements in hind limb function, they were unable to recover weight bearing capacity (Lu et al., 2002) and other neuronal populations, such as those of the corticospinal tract, failed to regenerate. Recently, Barbour et al. (2013) transplanted OEG and SCs sub-acutely, at 2 weeks, following spinal cord contusion in rat and reported an improvement in locomotor function that was accompanied by the growth of supraspinal projections originating from brainstem serotonergic regions.

The transplantation of genetically modified SCs, to secrete brain derived neurotrophic factor (BDNF; Menei et al., 1998) or D15A (Golden et al., 2007; Flora et al., 2013), a bifunctional neurotrophic molecule capable of activating both TrkB and C receptors, in transection or contusion SCI, respectively, have been shown also to promote considerable serotonergic axon regeneration at, and/or beyond, the injury level. Similarly, we have shown that genetic engineering of SCs, to enhance surface polysialic acid (PSA), also enhances their ability to support serotonergic growth across the lesion site and improve locomotor function when the SCs are transplanted into the contused spinal cord of rats (Ghosh et al., 2012). Recent work by Hodgetts et al. (2013) has reported that the transplantation of human mesenchymal precursor cells (Stro-1^+^), obtained from SCI patients can improve functional recovery and tissue sparing in athymic nude rats subjected to a 200 kDyne contusion injury at the vertebral level of T9-T10. The promotion of significant serotonergic axon growth within and surrounding the transplanted cells was a key anatomical change, although evidence of these 5-HT axons reaching the CPG in the caudal cord was not investigated.

The use of cell grafts, not only as a source of serotonergic neurons to provide direct input to the CPG but also as a substrate for supporting the growth of severed supraspinal serotonergic projections across the lesion to reinnervate the CPG, have thus shown potential for restoring locomotor function after SCI. Further enhancement of these approaches with regenerative factors to encourage greater axon growth into the caudal cord or with agonists of monoaminergic systems to enhance the functionality of caudal projections, may be able to provide more robust strategies for the recovery of function after SCI in which the spinal cord CPG remains intact.

## Conclusion

The serotonergic pathway works in concert with other monoaminergic systems in the synaptic control of motoneuron excitability. 5-HT contributes to the initiation of locomotion and other rhythmic motor behaviors as well as to the modulation of synaptic transmission in specific reflex pathways through its action on presynaptic terminals. Binding of 5-HT to specific receptor subtypes is responsible for initiating diverse intracellular signaling cascades that can alter the properties of ligand gated voltage-sensitive ion channels and produce a subsequent rapid depolarization of the postsynaptic membrane potential of target neurons to increase their intrinsic excitability. One of the major changes that occurs after SCI is the loss of most, if not all, of the descending monoaminergic input to the spinal cord below the level of the lesion, particularly to the spinal CPG, resulting in immediate and persistent locomotor dysfunction. Although axotomized serotonergic axons have the capacity to undergo sprouting after SCI, endogenous recovery of locomotor function is limited.

Substantial progress has been made in our understanding of the roles of the diverse number of 5-HT receptor subtypes and how they coordinate with one another and receptor signaling through other monoaminergic systems to alter motor output following SCI. The use of either pharmacological or cellular approaches to modulate the activity of various receptor subtypes in response to 5-HT or to provide 5-HT input caudal to the lesion site, respectively, have shown that the restoration of serotonergic signaling can be an effective means for promoting motoneuron excitability and restoring locomotor function after SCI. The use of genetic tools to investigate the role of specific 5-HT receptors in different aspects of motor output or in understanding the role of regenerating serotonergic axons in functional efficacy provided by different therapeutic modalities remains limited and should receive greater attention in future research.

Approaches to restore function after SCI through targeting 5-HT have shown some degree of success, however, a number of limitations remain to be resolved and questions answered. Though the use of 5-HT or specific 5-HT receptor agonists can stimulate CPG neuronal networks and induce locomotion, it is not clear whether such an approach can be employed for sustained functional recovery in light of receptor desensitization and/or loss of responses to the stimuli over time, a common phenomenon with GPCR family members. One strategy for achieving a sustained functional improvement using physiological levels of 5-HT could be through cellular therapies, where serotonergic neurons generated from various sources could be used to replace lost supraspinal serotonergic projections to the CPG below the level of SCI. Not only does a cell therapeutic approach eliminate the risk of side effects associated with systemic administration of high levels of 5-HT receptor agonists but also allows intraspinal delivery of 5-HT directly to where it is required after potentially a single intervention. Alternatively, the use of cell transplants, in combination with pro-regenerative therapies, can be used to bridge the injury site and provide a substrate for severed serotonergic axons to re-grow to their original caudal targets. Though the robustness and applicability of such approaches to a variety of SCI paradigms and injury levels has not yet been thoroughly investigated and many cell transplantation strategies to date have failed to promote the growth of serotonergic axons in significant numbers beyond the lesion into the contiguous caudal cord let alone beyond to the lumbar CPG. Another issue with cell therapeutic approaches is their limited long-term cell survival following transplantation due to the inflammatory environment of the injured spinal cord and rejection, potentially reducing their effectiveness in maintaining functional improvements over time. Therefore, extensive optimization of various cell therapeutic approaches is currently a priority in the field to maximize their therapeutic efficacy.

Another promising strategy to induce locomotor function after SCI in combination with 5-HT or its receptor agonists involves the activation of spinal locomotor networks via EES and data so far are encouraging, both in experimental SCI paradigms and clinically in complete SCI individuals. Similar to the use of pharmacological approaches alone is whether such a combination strategy can be implemented for the persistent restoration of function and whether additional interventions, including cell transplants, can enhance or prolong the benefits of this strategy. Further research at the molecular, cellular and functional level to trigger the necessary sequence of neurological events for complete restoration of locomotor function after human SCI continues to remain as a major challenge and a research area of primary focus. The promotion or facilitation of serotonergic signaling, so as to enhance motoneuron excitability, stimulate CPG activity and restore locomotor function, is one direction at the forefront of research for generating putative interventional approaches for the treatment of SCI.

## Conflict of interest statement

The authors declare that the research was conducted in the absence of any commercial or financial relationships that could be construed as a potential conflict of interest.
